# Probiotics at War Against Viruses: What Is Missing From the Picture?

**DOI:** 10.3389/fmicb.2020.01877

**Published:** 2020-08-20

**Authors:** Santosh Kumar Tiwari, Leon M. T. Dicks, Igor V. Popov, Alena Karaseva, Alexey M. Ermakov, Alexander Suvorov, John R. Tagg, Richard Weeks, Michael L. Chikindas

**Affiliations:** ^1^Department of Genetics, Maharshi Dayanand University, Rohtak, India; ^2^Department of Microbiology, Stellenbosch University, Stellenbosch, South Africa; ^3^Center for Agro-Biotechnology, Faculty of Bioengineering and Veterinary Medicine, Don State Technical University, Rostov-on-Don, Russia; ^4^Institute of Experimental Medicine, Saint Petersburg, Russia; ^5^Saint Petersburg State University, Saint Petersburg, Russia; ^6^BLIS Technologies Ltd., Dunedin, New Zealand; ^7^Health Promoting Naturals Laboratory, School of Environmental and Biological Sciences, Rutgers, The State University of New Jersey, Brunswick, NJ, United States

**Keywords:** probiotics, bacteriocins, gut microbiota, antiviral, immunity, viruses, SARS-CoV-2, COVID-19

## Abstract

Our world is now facing a multitude of novel infectious diseases. Bacterial infections are treated with antibiotics, albeit with increasing difficulty as many of the more common causes of infection have now developed broad spectrum antimicrobial resistance. However, there is now an even greater challenge from both old and new viruses capable of causing respiratory, enteric, and urogenital infections. Reports of viruses resistant to frontline therapeutic drugs are steadily increasing and there is an urgent need to develop novel antiviral agents. Although this all makes sense, it seems rather strange that relatively little attention has been given to the antiviral capabilities of probiotics. Over the years, beneficial strains of lactic acid bacteria (LAB) have been successfully used to treat gastrointestinal, oral, and vaginal infections, and some can also effect a reduction in serum cholesterol levels. Some probiotics prevent gastrointestinal dysbiosis and, by doing so, reduce the risk of developing secondary infections. Other probiotics exhibit anti-tumor and immunomodulating properties, and in some studies, antiviral activities have been reported for probiotic bacteria and/or their metabolites. Unfortunately, the mechanistic basis of the observed beneficial effects of probiotics in countering viral infections is sometimes unclear. Interestingly, in COVID-19 patients, a clear decrease has been observed in cell numbers of *Lactobacillus* and *Bifidobacterium* spp., both of which are common sources of intestinal probiotics. The present review, specifically motivated by the need to implement effective new counters to SARS-CoV-2, focusses attention on viruses capable of co-infecting humans and other animals and specifically explores the potential of probiotic bacteria and their metabolites to intervene with the process of virus infection. The goal is to help to provide a more informed background for the planning of future probiotic-based antiviral research.

## Introduction

By the end of July 10, 2020, the SARS-CoV-2 virus (severe acute respiratory syndrome coronavirus 2), responsible for COVID-19 (coronavirus disease 2019) infections, had clinically affected more than 12.3 million people in 215 countries (Worldometer, 2020)^1^. Of those infected, approximately 7 million recovered, more than 557,491 died, and approximately 4.6 million were considered to be active cases and were still fighting the infection. Coronaviruses are enveloped, positive-sense RNA viruses with spiky projections on their surface, giving the particles a crown-like appearance under an electron microscope, hence the name coronavirus (Richman et al., 2016). The RNA genome of coronaviruses (CoVs) is amongst the largest of all viruses (Belouzard et al., 2012) and is susceptible to mutations giving rise to new strains with altered virulence (Hilgenfeld, 2014). Thus far, seven strains of human CoVs have been identified: 229E, NL63, OC43, HKU1, Middle East respiratory syndrome (MERS)-CoV, severe acute respiratory syndrome (SARS)-CoV, and 2019-novel coronavirus (nCoV) (Chang et al., 2016; Paules et al., 2020). Of these, SARS-CoV, MERS-CoV, and 2019-nCoV are the most infectious and have caused several CoV outbreaks (Paules et al., 2020). Symptoms of COVID-19 are associated with acute respiratory restrictions, cough, sore throat, and fever, followed by organ failure and often secondary infections (Chen et al., 2020). These symptoms are linked to an increase in the levels of cytokines IL1, IL7, IL 10, GCSF, IP10, MCP1, MIP1A, and TNFα (Chen et al., 2019). A strong correlation has been reported between disease progression and the composition of the gut microbiota. Some patients with COVID-19 showed intestinal microbial dysbiosis, with a decrease in cell numbers of *Lactobacillus* and *Bifidobacterium* (Xu et al., 2020). The authors suggested that the use of probiotics, along with prebiotics, might help normalize the stability of the intestinal microbiota and lower the risk of secondary infections. Animals are one of the most important “constituents” of the human ecosystem, and as such, they must be considered as a primary source, or incubator, of novel viral diseases and outbreaks in humans. A particularly pertinent example of this is the COVID-19 pandemic. In this review, we focus upon the antiviral potential of lactic acid bacteria (LAB) probiotics and their secondary metabolic compounds and discuss the transfer of viruses between animals and humans. Current and past research efforts into the use of probiotics and/or their metabolites to combat viral infections are sorely lacking. As such, an objective of this review is to not only discuss these research efforts, but also to use this information to outline a roadmap that may be used to help structure and focus future research efforts toward the use of probiotics in the control of viral infections.

## Probiotic-Produced Bacteriocins for the Control of Microbial Infections

Probiotics are defined as “live microorganisms that, when administered in adequate amounts, confer a health benefit on the host” (Hill et al., 2014). Most of the probiotics that have been reported and marketed to-date are LAB having the ability to inhibit certain pathogens at various body sites. While some probiotics are targeted for animal use, the majority have principally been utilized to benefit human health via their effect on the composition of the gut microbiota (Hill et al., 2014). Applications include remediation of dysbiosis within the gut microbiota and the more specific treatment and prophylaxis of bacterial and fungal infections. The mode of action of probiotics for the therapeutic treatment of bacterial and fungal infections is well documented (Stoyanova et al., 2012; Suvorov, 2013). There are, however, also reports indicating the successful application of probiotics in the treatment of various intestinal, respiratory, and urogenital diseases caused by viruses. Such antiviral probiotics might be a preferable alternative to more conventional antiviral agents due to the escalating emergence of viruses that are resistant to commonly used antivirals (WHO, 2008). Suggested primary mechanisms of probiotic action against viruses include direct probiotic cell interaction with the targeted viruses, production of antiviral metabolites and modulation of the eukaryotic host’s immune system (Ichinohe et al., 2011; Wu et al., 2013; Al Kassaa et al., 2014; Drider et al., 2016). Other possibilities range from modulation of the host microbiota to interaction with eukaryotic epithelial cells and effects on the electrolyte potential (Olaya Galán et al., 2016).

Virtually all bacteria, including the LAB probiotics, are known to produce ribosomally synthesized substances of proteinaceous nature that exhibit bactericidal activity. These substances are collectively called bacteriocins (Alvarez-Sieiro et al., 2016). Many bacteriocins have been extensively studied, and some have been commercially developed due to their ability to preserve food and to exhibit therapeutic antimicrobial activity. Many bacteriocins are extremely thermostable and are active over a broad pH range. Furthermore, most bacteriocins are non-immunogenic, and are generally colorless, odorless, and tasteless. These characteristics make bacteriocins particularly attractive for food preservation and health care applications (for reviews see: Alvarez-Sieiro et al., 2016). Due to the widespread emergence of resistance to most of the commonly used therapeutic antibiotics (Woolhouse et al., 2016), new classes of antimicrobial agents are desperately being sought. Tagg et al. (1976) have defined bacteriocins as peptides active against closely related bacteria. However, some of the more recently described bacteriocins are also active against relatively distantly related bacteria (Gupta et al., 2016; Belguesmia et al., 2020). The sensitivity of a target bacterium to bacteriocins depends on the physico-chemical characteristics of the environment, of which pH, ionic strength, and the presence of neutralizing or membrane-disrupting molecules all play a major role (Belguesmia et al., 2010).

Probiotic bacteria often rely on bacteriocins to compete with other bacteria and to colonize a niche, such as in the gastrointestinal tract (Dobson et al., 2011a). While the intestinal microbiota comprises a dynamic community and plays an integral role in gut health, the bacteriocin thuricin CD inhibited the growth of *Clostridium difficile* in a colon model, without having a significant effect on the remainder of the microbiota (Rea et al., 2011). Similarly, a bacteriocin produced by the probiotic strain *Lactobacillus salivarius* UCC118 protected mice against infection caused by *Listeria monocytogenes* (Corr et al., 2007). Other studies have shown that modulation of the gut microbiota by bacteriocins may lead to an increase in body mass (Murphy et al., 2013).

Metabolites such as lactic acid, hydrogen peroxide, and also bacteriocins produced by LAB, have been studied for their ability to decrease viral loads (Al Kassaa et al., 2014; Drider et al., 2016; Chikindas et al., 2018). Other studies have shown that bacteriocins may also play an important role in host defense and cell signaling (Cotter et al., 2005). These antimicrobial proteins (AMPs) have a beneficial effect on the host microbiota and various organs of the body, and have shown promise in controlling potential pathogens (for reviews see: Drider et al., 2016; Chikindas et al., 2018). The antibacterial activity of bacteriocins is relatively well understood, while the basis for their antiviral activities has received far less attention and is only now being studied in depth. Wachsman et al. (2000) reported that a peptide produced by *Enterococcus faecium* CRL35 inhibited the late stages of HSV-1 and HSV-2 replication (Wachsman et al., 2000). In addition, Serkedjieva et al. (2000) demonstrated that a 5.0 kDa-bacteriocin produced by *Lactobacillus delbrueckii* subsp. *bulgaricus*, highly specifically inhibited replication of influenza virus A/chicken/Germany, strain Weybridge (H7N7) and strain Rostock (H7N1). Expression of the viral glycoproteins neuraminidase, hemagglutinin, and nucleoprotein on the surface of infected cells, virus-induced cytopathic effect, infectious virus yield, and hemagglutinin production were all reduced at concentrations of the peptide that were non-toxic for eukaryotic cells (Serkedjieva et al., 2000). It would be interesting to determine if there is any relation between the antiviral activity of a bacteriocin and viral structure, e.g., naked versus enveloped. To the best of our knowledge, information on this relationship has not been published and any conclusions would be speculative. This knowledge gap merits further research.

Several other bacteriocins have been found to be active against viruses. Enterocin AAR-74 partially reduced viral replication, while enterocin AAR-71 and erwiniocin NA4 completely eliminated replication (Qureshi H. et al., 2006). Enterocins CRL35 and ST4V were active against strains of HSV-1 and HSV-2 in Vero and BHK-21 cells, inhibiting the late stages of viral replication (Wachsman et al., 1999; Todorov et al., 2005). This antiviral activity of bacteriocins could be due to their blocking of receptor sites on host cells (Wachsman et al., 2003). Labyrinthopeptin A1 (LabyA1) is a carbacyclic lantibiotic which exhibited consistent and broad anti-HIV activity against cell-line adapted HIV-1 strains (Férir et al., 2013). LabyA1 showed very consistent anti-HIV-1 activity against nine clinical isolates of HIV-1 and repressed intercellular transmission between HIV-infected T cells and uninfected CD4+ T cells (Férir et al., 2013). This inhibited the transmission of HIV from DC-SIGN+ cells to uninfected CD4+ T cells. Synergistic anti-HIV-1 and anti-HSV-2 activity was confirmed using LabyA1 in dual combination with the antiretroviral agents raltegravir, acyclovir tenofovir, enfuvirtide, and saquinavir (Al Kassaa et al., 2014). Remarkably, labyrinthopeptins A1 and A2 were also able to inhibit the entry of the human respiratory syncytial virus (hRSV) into human carcinoma-derived lung cells *in vitro* (Haid et al., 2017).

Lange-Starke et al. (2014) demonstrated Inhibitory activity against murine norovirus (MNV) associated with a mixture of complex metabolites present in the cell-free supernatants (CFS) of *L. curvatus* 1. This bacterial strain-specific antiviral effect was subsequently confirmed in five trials (Botic et al., 2007). Further studies were conducted to assess the antiviral properties of the CFS. Since no significant antiviral activity was present following heat treatment, it was suggested that the antiviral substance was of proteinaceous nature and possibly a bacteriocin. Potential interference by the active agent with virus replication or cell adsorption was suggested. In another study, Serkedjieva et al. (2000) found that bacteriocin B1 from *L. delbrueckii* inhibits some intracellular virus replication steps. To date, no bacteriocin activity against SARS-CoV-2 has been reported, suggesting further exploration of this possibility is warranted.

## Bacteriocin-Producing Probiotics for Combating/Prophylaxis of Viral Infections

### Bacteriocins and Modulation of the Gut Microbiota

The assumed ability of bacteriocins to alter the gut microbiota by targeting detrimental components without having a negative influence on the beneficial microorganisms is an idealistic concept. The eukaryotic host is colonized by trillions of microbes in symbiotic association, and some of these microbes have the potential to become pathogenic during dysbiosis (Turnbaugh et al., 2007; Round and Mazmanian, 2009). The disruption of the intestinal microbiota due to antibiotic treatment can trigger pathogenic behavior in some bacteria, which may result in their overgrowth within the host’s commensal microbiota. Several reports have described a high incidence of bacteriocin production among enterococci. A large percentage of enterococci isolated from stool specimens do produce bacteriocins. Enterococci isolated from infections also frequently produce bacteriocins, such as bacteriocins 31 and 41 from *Enterococcus faecalis* (Tomita et al., 1996, 2008) and bacteriocins 43 (Todokoro et al., 2006), 32 (Nes et al., 1996), and 51 (Yamashita et al., 2011) from *E. faecium*.

Kommineni et al. (2015) suggested that bacteriocins produced by enterococci play a role in colonization of the GI tract. Bacteriocin production may enhance the stability of enterococcal communities, fostering their competition with closely related species and promoting the growth of the bacteriocin-producing strains (Corr et al., 2007; Hibbing et al., 2010). It was found that *E. faecalis* producing bacteriocin 21 were able to better colonize the mouse GI tract than were bacteriocin non-producers. To test if bacteriocin-21, encoded by genes on plasmid pPD1, facilitates colonization by enterococci, the authors introduced an in-frame deletion of bacAB into pPD1. The bacteriocin-depleted mutant lost its colonization advantage over wild-type *E. faecalis*. Colonization experiments with a bacteriocin 21 producer showed it to become more abundant in the feces and throughout the GI tract, suggesting more effective colonization (Kommineni et al., 2015). The expression of bacteriocins by commensal bacteria may have a beneficial effect on their competition for niches within the GI tract. Bacteriocins produced by commensal strains in a specific niche may thereby also have a therapeutic role and may prevent the growth of multidrug-resistant bacteria without disrupting the indigenous microbiota. The introduction of bacteriocin-producing commensal strains to the GI tract could be a method for the removal of antibiotic-resistant enterococci from the GI tract of patients and may also help in preventing the re-emergence of enteric infections (Kommineni et al., 2015). The majority of studies to date have shown that bacteriocin-producing strains have the ability to inhibit the proliferation of well-established gut pathogens. Bacteriocins may thus be used to either prevent or treat infections (Hegarty et al., 2016).

Among other significant commensals, LAB play a vital role in keeping the human and animal gut microbiome in a balanced state (Gillor et al., 2008; Dicks and Botes, 2010; Mackowiak, 2013) and in maintaining the integrity of the gut wall (for reviews see Bajaj et al., 2015; Dicks et al., 2018). Other probiotic attributes include stimulation of the immune system, reduction of lactose, production of vitamins (B and C), prevention of colon cancer and Crohn’s disease, and reduction of cholesterol (reviewed by Dicks and Botes, 2010).

Probiotic LAB produce a wide variety of antimicrobial compounds ranging from the metabolites hydrogen peroxide, short-chain fatty acids, and lactic acid to bacteriocin-like inhibitory substances (BLIS) and bacteriocins (Dobson et al., 2011a). In this review, we are focused primarily on LAB probiotics and their bacteriocins, although it is commonly accepted that virtually all bacteria produce bacteriocins as defensive weaponry and as communication signals. Despite the many papers published on bacteriocin structures, functions, and food applications, unforgivably little research has been devoted to the medicinal properties of these peptides, including their activity against eukaryotic viruses, a feature most certainly of relevance for health promotion (Dicks et al., 2011; Chikindas et al., 2018). The reason for this is largely due to the perception that bacteriocins have limited stability in the GIT, serum, liver, and kidneys (Joerger, 2003; McGregor, 2008). Although bacteriocins are typically degraded by proteolytic enzymes, some exceptions to the rule have been reported. When nisin F was injected into the peritoneal cavity of mice, it remained active against *Staphylococcus aureus* (*in vivo*) for 15 min (Brand et al., 2010). Nisin A (Bartoloni et al., 2004) and mutacin B-Ny266 (Mota-Meira et al., 2000) survived conditions in the peritoneal cavity, albeit for short periods. Microbisporicin remained active for a few minutes when intravenously administered to mice (Castiglione et al., 2008). Lacticin 3147 prevented the systemic spread of *S. aureus* Xen 29 in mice and thuricin CD, a two-peptide bacteriocin produced by *Bacillus thuringiensis* 6431 (although not a LAB), inhibited the growth of *Clostridium difficile in vivo* (Rea et al., 2010). When ingested, nisin F had a stabilizing effect on the bacterial population in the murine GIT (Van Staden et al., 2011). *Enterococcus mundtii* produced bacteriocin ST4SA when cells were exposed to conditions simulating the GIT (Granger et al., 2008). Plantaricin 423, produced by *Lactobacillus plantarum*, was expressed when cells were exposed to simulated gastric fluid (Ramiah et al., 2007). Nisin F, produced by *Lactococcus lactis*, prevented respiratory tract and subcutaneous skin infections instigated by *S. aureus* (De Kwaadsteniet et al., 2009, 2010).

While on the subject of bacteriocins, it should be made clear that these proteinaceous, ribosomally synthesized antimicrobials of bacterial origin are significantly different from conventional therapeutic antibiotics (Weeks and Chikindas, 2019). Unlike antibiotics, strains sensitive to bacteriocins seldom develop resistance, and the mechanisms of resistance are different from those reported for antibiotics (for review see de Freire Bastos et al., 2015). Strains that do develop resistance undergo major structural changes in their cell walls and cell membranes. *L. monocytogenes* developed resistance to nisin after altering the fatty acid and phospholipid composition in its cell membrane (Abee et al., 1995). *S. aureus* and *Bacillus subtilis* increased the D-alanyl ester and galactose in their cell walls (Peschel et al., 1999; Vadyvaloo et al., 2002; Chatterjee et al., 2005). Some strains of *S. aureus* prevented nisin from entering the cell by developing a mutation in the *nsaS* (nisin susceptibility-associated sensor) (Blake et al., 2011; Collins et al., 2012). Other Gram-positive strains developed thicker cell walls, which prevented nisin from docking with lipid II (Mantovani and Russell, 2001). *L. monocytogenes* gained resistance to mesentericin Y105 by inactivating the *rpoN* gene that encodes the σ^54^ subunit of bacterial RNA polymerase (Robichon et al., 1997). Some *Bacillus* spp. produced non-proteolytic nisin-inactivating enzymes that reduced dehydroalanine (Jarvis, 1967; Jarvis and Farr, 1971). Other forms of resistance described have been less structure-based, such as developing a requirement for Mg^2+^, Ca^2+^, Mn^2+^, and Ba^2+^ (Ming and Daeschel, 1993; Mazzotta and Montville, 1997; Crandall and Montville, 1998). Resistance may also be pH related. In an acidic environment, *L. lactis* repelled nisin by binding high concentrations of the peptide to its cell surface (Hasper et al., 2006).

### Bacteriocins and Immune Modulation Against Viral Infections

Modulation of the immune system is commonly considered a valid approach to the prophylaxis of viral infections, including COVID-19 (for review, see Jayawardena et al., 2020). The immunomodulatory properties of LAB are well documented. Intestinal strains have the ability to alter the roles of dendritic cells (DC), monocytes/macrophages, and T and B lymphocytes, thereby enhancing the phagocytosis of pathogenic bacteria (Grasemann et al., 2007). *In vitro* studies have shown that LAB induce the release of the pro-inflammatory cytokines TNF-α and IL-6, thus stimulating non-specific immunity (Isolauri et al., 2001). *Lactobacillus rhamnosus* GG increased the number of rotavirus-specific IgM secreting cells in infants who had been administered an oral rotavirus vaccine (Cross, 2002). Feeding mice with *Lactobacillus casei* Shirota prior to an influenza virus challenge protected the upper respiratory tract significantly (Cross, 2002). Yogurt supplemented with *Lactobacillus acidophilus*, *Bifidobacterium infantis*, and *Bifidobacterium bifidum* enhanced mucosal and systemic IgA responses to cholera toxin in mice (Kaur et al., 2002). As expected, most of reports have focused on species of LAB in the human GIT. It would be interesting to determine if transient strains of LAB have the same effect on the immune system.

Surprisingly, only a few reports have been published on the immunomodulatory properties of bacteriocins (Brand et al., 2013; Kindrachuk et al., 2013). This is an important area that requires further investigation, especially since one of the first studies (Kindrachuk et al., 2013) had shown that the immunomodulatory properties of nisin are superior to that reported for the human cationic peptide LL-37 (Scott et al., 2002). Nisin, gallidermin, and Pep5 induced the discharge of multiple chemokines at concentrations similar to those recorded for LL-37 (Scott et al., 2002). Mice pre-treated with nisin, and then infected with *Escherichia coli* and *Salmonella typhimurium*, showed a significant reduction in bacterial cell numbers compared to the control group (Kindrachuk et al., 2013). Since nisin is inactive against Gram-negative bacteria, protection against these pathogens was ascribed to increased immunity. Contradictory findings were reported by Brand et al. (2013). These authors did not observe a noticeable immune response with continuous *in vivo* injection of nisin F into the peritoneal cavity of mice. The activity of interleukin-6, interleukin-10, and tumor necrosis factor was stimulated, irrespective of treatment with active or inactive nisin F. However, the overall immune response was not high enough to trigger an abnormal increase in antigenic immune reactions (Brand et al., 2013).

Begde et al. (2011) reported the toxic side effects of a sample containing a combination of nisins A and Z against human lymphocytes and neutrophils. The administration of Nisaplin (a commercial form of nisin A) to mice for 30 and 75 days resulted in an increase in CD4 and CD8 T-lymphocytes, but a decrease in B-lymphocytes (De Pablo et al., 1999). However, no side effects were recorded after 100 days of administration. Enhanced phagocytic activity of peritoneal cells was observed after long-term administration of Nisaplin. In another study, treatment with nisin resulted in immunostimulation of head kidney macrophages in fish (Villamil et al., 2003). Nisin, purified by RP-HPLC and vaginally administered to rats, was not toxic to host cells (Gupta et al., 2008). Nisin, pediocin, and peptide AS-48 have all shown immunogenic properties in antibody studies (Maqueda et al., 1993; Suárez et al., 1996; Martinez et al., 1997).

Ancovenin, a cinnamycin-like lantibiotic, inhibits angiotensin I converting enzyme (ACE), which plays an important role in regulating blood pressure through the conversion of angiotensin I to angiotensin II (Kido et al., 1983). Cinnamycin-like lantibiotics also inactivated phospholipase A2 by sequestering phosphatidylethanolamine (the substrate for phospholipase A2), thereby indirectly mediating inflammatory responses (Märki et al., 1991; Zhao, 2011). Phospholipase A2 plays a role in the release of arachidonic acid. The latter is oxidized to eicosanoids, such as prostaglandins and leukotrienes, serving as strong mediators of the immune system. Most higher eukaryotic species, such as mammals, have CAMPs (cationic antimicrobial peptides) interacting with the innate immune system. These peptides are usually positively charged, relatively small, and hydrophobic, which are also typical characteristics of lantibiotics (Sahl et al., 2005). It is thus not surprising that many researchers are advocating the use of CAMPs (including bacteriocins) as an alternative to antibiotics, especially for the killing of multidrug-resistant strains (Behrens et al., 2017). Bacteriocins modulate interleukin production (*in vivo*) and trigger the production of CD4(+) and CD8(+) T cells (Malaczewska et al., 2019). Modulation of the immune system can protect eukaryotic cells against viral infections, as observed in studies conducted on poultry (Yitbarek et al., 2018). Bacteriocin producing probiotic strains could thus protect humans and other animals against viral infections, including Covid-19.

Dreyer et al. (2019) were the first to show *in vitro* that bacteriocins can cross the gut-blood barrier (GBB). The authors showed that 85% of plantaricin 423, 75% of nisin, and 82% of bacST4SA (a class IIa bacteriocin produced by *E. mundtii*) migrated across a Caco-2 cell monolayer within 3 h. In the case of HUVEC cells, 93% plantaricin 423, 88% nisin, and 91% bacST4SA migrated across the cell monolayers within 3 h (Dreyer et al., 2019). Further research has to be done to determine the rate at which bacteriocins (either active or inactive) cross the GBB if they have the ability to re-enter tissue cells and if so, accumulate in organs. Dreyer et al. (2019) concluded that the rate at which bacteriocins cross cell membranes most likely depends on the physiological and biochemical state of the membrane. The authors have also shown that class IIa bacteriocins retained a higher level of antibacterial activity compared to class I bacteriocins (lantibiotics). More bacteriocins will have to be studied to confirm these findings. It would also be interesting to determine the effects that molecular size, the number of sulfide bridges (folding of the peptide), hydrophobicity, and charge have on the migration of bacteriocins across epithelial cell membranes.

### Antiviral Properties of Probiotic Bacteriocins

The first reports of LAB inactivating viruses were published around 30 years ago. At that time, antiviral activity was mostly ascribed to the protein denaturing reactions of hydrogen peroxide and lactic acid produced by *Lactobacillus* spp., leading to the inactivation of the human immunodeficiency virus type 1 (HIV-1) and human simplex virus type 2 (HSV-2) (Martin et al., 1985; Klebanoff and Coombs, 1991; Tuyama et al., 2006; Conti et al., 2009). In other studies, a non-protein cell wall component of *Lactobacillus brevis* reduced the replication of HSV-2 (Mastromarino et al., 2011). Probiotic strains of *Lactobacillus paracasei*, *Lactobacillus paracasei* subsp. *rhamnosus*, *L. plantarum*, and *Lactobacillus reuteri* entrapped vesicular stomatitis viruses by adhering to the particles (Botic et al., 2007). Similar modes of action were reported for the inhibition of influenza viruses by *E. faecium* NCIMB 10415 (Wang et al., 2013) and for the inhibition of HSV-2 by *L. gasseri* CMUL57 (Al Kassaa et al., 2014). Since then, a large number of reports have been published on the antiviral properties of LAB (reviewed by Al Kassaa et al., 2014). Proposed modes of antiviral action include direct interaction between the LAB and viruses (Botic et al., 2007; Wang et al., 2013; Al Kassaa et al., 2014), production of antiviral substances (Martín et al., 2010; Maragkoudakis et al., 2010; Mastromarino et al., 2011), and stimulation of the host’s immune system (Szajewska and Mrukowicz, 2001; Yasui et al., 2004; De Vrese et al., 2006; Olivares et al., 2007; Boge et al., 2009; Rautava et al., 2009; Kawashima et al., 2011; Salva et al., 2011; Villena et al., 2011; Khania et al., 2012; Kiso et al., 2013).

*Lactobacillus plantarum* L-137 decreased the levels of influenza virus H1N1 in infected mice by eliciting a pro-inflammatory response (Maeda et al., 2009). Similar findings were reported for *Lactobacillus fermentum* CECT5716 and *L. casei* DN114-001. Both strains stimulated the formation of antibodies to H1N1 (Olivares et al., 2007; Boge et al., 2009). A combination of *L. gasseri* PA 16/8, *Bifidobacterium longum* SP07/3, and *B. bifidum* MF 20/5 reduced symptoms of the common cold (De Vrese et al., 2006), *L. rhamnosus* GG reduced the incidence of respiratory virus infections (Rautava et al., 2009) and treatment with *L. acidophilus* strain NCFM reduced influenza-like symptoms (Leyer et al., 2009). These are all promising findings and warrant further research.

Most reports on the antiviral activities of LAB have focused on class IIa bacteriocins, including enterocin AAR-71 and enterocin AAR-74 from *E. faecalis* (Qureshi H. et al., 2006), enterocin ST5Ha from *E. faecium* (Todorov et al., 2010), enterocin ST4V and enterocin CRL35 from *E. mundtii* (Wachsman et al., 2003; Todorov et al., 2005), and a peptide designated by the authors as a “bacteriocin” from *L. delbrueckii* (Serkedjieva et al., 2000). Enterocin AAR-74 reduced the proliferation of coliphage HSA 10-fold, whereas enterocin AAR-71 had no effect on phage HSA (Humaira et al., 2006). Enterocin ST4V inhibited herpes viruses HSV-1 and HSV-2 in a dose-dependent manner (Todorov et al., 2005). Although enterocin CRL35 inhibits late stages of HSV-1 and HSV-2 replication, the mode of action of enterocin ST5Ha is uncertain. Interestingly, the non-LAB bacteriocin subtilosin A also inhibited HSV-1, its drug-resistant mutant, and HSV-2A, most likely acting at the late stage of virus replication. However, subtilosin A did not act on non-enveloped viruses (Torres et al., 2013; Quintana et al., 2014). Enterocins CRL35 and ST4V acted on the multiplication of virus particles (Wachsman et al., 1999, 2003; Todorov et al., 2005). Small differences in amino acid sequence have seemingly had a huge effect on antiviral activity, as reported for enterocin CRL35. A derivative of enterocin CRL35, missing two cysteine residues, was inactive against herpes viruses (Salvucci et al., 2007). The same derivative was also inactive against bacteria. This, however, does not necessarily mean that the same peptide segment is responsible for both anti-bacterial and antiviral activities. The carbacyclic lantibiotic labyrinthopeptin A1 (LabyA1) inactivated the HIV virus and prevented its transmission between CD4 cells (Férir et al., 2013). A bacteriocin produced by *L. delbrueckii* subsp. *bulgaricus* 1043 inhibited one of the influenza viruses (Serkedjieva et al., 2000). Thus far, most reports on bacteriocins having antiviral activity have been based on observations of inhibition of virus replication. Further research is now needed to elucidate the exact mode of antiviral activity.

Little is known about the pharmacodynamics of bacteriocins, including their interactions with eukaryotic cells and individual cellular components. We know probiotic LAB trigger anti-inflammatory responses in the innate immune system by signaling dendritic cells (DCs) to secrete anti-inflammatory cytokines such as interleukin 10 (IL-10) (de LeBlanc et al., 2011). Probiotics can also down-regulate pro-inflammatory cytokines by interfering with inflammatory signaling pathways such as the nuclear factor-kappa B (NF-κB) and mitogen-activated protein kinase (MAPK) pathways (Yoon and Sun, 2011). Activation of these pathways increases the secretion of pro-inflammatory cytokines that may lead to damage of intestinal epithelial cells. It is not known if bacteriocins have the same effect on the immune system.

## Delivery of Bacteriocins to the Host

Prevention of viral infections would be best managed via either intravenous injections or oral administration. An orally administered agent would have to survive the harsh conditions in the GIT and then cross the highly selective GBB. Irrespective of the method of administration, the antiviral agent would have to be resistant to proteolytic enzymes in the GIT and bloodstream. Other hurdles include binding to plasma proteins, red blood cells, ions, etc. Protection against degradation is technically possible by encapsulating the antiviral agent with specific polymers to form nanoparticles (Heunis et al., 2010). By coating the surface of the particle with selected ligands, the antiviral agent could be delivered to a specific organ or infected tissue. Probiotic bacteria, in natural form or encapsulated, may be used as delivery vehicles to secrete antiviral agents in the GIT (Marteau and Shanahan, 2003; Heunis et al., 2010). With recent developments in nanotechnology (Pathak, 2009; Soares et al., 2016; Ragelle et al., 2017) and drug delivery, targeted delivery of antiviral agents could become the norm. To use probiotic cells as target-specific delivery agents, we first need to understand the effects GIT conditions have on the proliferation of probiotic bacteria and on the regulation of their genes.

Nanotechnology provides a means of protecting bacteriocins from both the immune system and proteolytic enzymes. The first experiments on the encapsulation of peptides were done with liposomes. In subsequent studies, peptides were encapsulated into natural hydrogels (e.g., chitosan, fibrin, collagen, gelatin, dextran, hyaluronic acid, and alginate). Experiments have also been done with dextran and synthetic polymers such as poly(ε-caprolactone) (PCL), poly(ethylene oxide) (PEO), poly(D,L-lactide-*co*-glycolide) (PLGA), poly(lactic acid) (PLA), poly(vinyl alcohol) (PVA), polyphosphazene poly(acrylic acid) (PAA) and poly(*N*-isopropylacrylamide) (PNIPAAm). For a review on encapsulation with these polymers, the reader is referred to Lee and Yuk (2007). Anticancer and antipsychotic drugs, insulin, and hormones (estradiol and tetanus toxoid) have all been successfully encapsulated using bPLGA (Mundargi et al., 2008; Kumari et al., 2010). In the body, PLGA is hydrolyzed to lactic and glycolic acid and results in minimal systemic toxicity (Di Toro et al., 2004). PLA, an eco-friendly polymer, is used to encapsulate antipsychotic drugs (e.g., savoxepine), oridonin (a natural diterpenoid), restenosis drugs (e.g., tyrphostins), hormones (e.g., progesterone) and bovine serum albumin (BSA) (Leo et al., 2004; Kumari et al., 2010). Less biodegradable polymers, such as PCL, are used in the manufacturing of long-term implants. Examples include tamoxifen and taxol used in the treatment of cancer, and insulin used to treat diabetes (Kumari et al., 2010). Insulin, cyclosporine A, and antihormonal drugs such as glycyrrhizin have been encapsulated in chitosan. The anticancer drug paclitaxel, anti-HIV drug didanosine, antimalarial drug chloroquine phosphate, oligonucleotides, and BSA have been encapsulated using gelatin (Kumari et al., 2010).

With the latest developments in nanotechnology, it is now possible to design nanoparticles that are small enough to pass through epithelial cells in the GIT and able to circulate in the bloodstream long enough to deliver bacteriocins to the site of infection. Site-directed delivery would not only reduce the inhibitory concentration required, but would also minimize possible toxic side effects. Chai et al. (2014) studied transport pathways across epithelial cell monolayers using a model system. By selectively inhibiting specific pathways, the authors studied individual transport mechanisms involved in the transcytosis of nanoparticles. For a review on the physicochemical properties of nanoparticles in endocytosis and transcytosis mechanisms, the reader is referred to des Rieux et al. (2006). The crossing of biological barriers depends on the tissue and blood circulation at the site of entrance, as pointed out by Brannon-Peppas and Blanchette (2004) and Feng (2004). Nanoparticles with hydrophobic properties are easily recognized by macrophages and destroyed (Kumari et al., 2010). By coating nanoparticles with hydrophilic polymers, their circulation in the bloodstream is enhanced, as is their stability (Brigger et al., 2002).

Nanoparticles are delivered either via passive or active targeting (reviewed by Wang and Thanou, 2010). Passive targeting is mainly used in delivery to tumor tissue (Kobayashi et al., 2014). Tumor tissue does not have a fully developed lymphatic system, and nanoparticles tend to accumulate inside the tumor. Active targeting can be directed toward any cell type, although most of the studies to date have focused on tumors. In this case, specific epitopes are needed on the surface of the target. Recognition sites are mimicked with specially “decorated” nanoparticles.

Peptides have been attached to carrier molecules such as polyethylene glycol (PEG) (Veronese and Mero, 2008; Pasut and Veronese, 2009; Jevsevar et al., 2010) or fatty acids (Avrahami and Shai, 2002). Most studies on the delivery of bacteriocins have been focused on the design of antimicrobial food packaging films. Only a few studies (Scannell et al., 2000; Cutter et al., 2001; Marcos et al., 2007; Malheiros et al., 2010) have been published on bacteriocin delivery systems for biomedical applications. The stability of nisin was improved by incorporation of its unique biosynthetic genes onto unrelated peptides with improved *in vitro* and *in vivo* activity and stability (Kuipers et al., 2006, 2008; Kluskens et al., 2009; Majchrzykiewicz et al., 2010; Moll et al., 2010). Nisin was further stabilized by introducing D-amino acids (Bessalle et al., 1990; Hong et al., 1999) in the amino acid ring structures, thereby increasing cyclization of the peptide (Li and Roller, 2002). Rink et al. (2010) combined the two approaches to create peptides having enhanced *ex vivo* stability.

Salmaso et al. (2004) encapsulated nisin in PLA nanoparticles by using semicontinuous precipitation in compressed CO_2_. The release of nisin from these nanoparticles depended on the salt concentration and the pH of the medium. Heunis et al. (2010) were the first to electrospin a class II bacteriocin, plantaricin 423, into PEO polymeric nanofibers. Slow and controlled release of plantaricin 423 was achieved by encapsulation in PDLA-PEO (Heunis et al., 2010). Nisin, electrospun into nanofibers of the same composition, reduced the cell numbers of *S. aureus* associated with skin infections (Heunis et al., 2014). Co-spinning of silver nanoparticles and 2,3-dihydroxybezoic acid into PDLA-PEO nanofibers increased the antimicrobial spectrum of nisin (Ahire and Dicks, 2015; Ahire et al., 2015). Subtilosin, incorporated into PVA nanofibers, inhibited the activity of the Herpes simplex virus type 1 (Torres et al., 2013). The high surface to volume ratio of nanofibers makes them ideal drug delivery vehicles and they may even be used in the slow and controlled release of growth factors, or incorporated into prostheses and wound dressings (Chew et al., 2005; Jiang et al., 2005; Kim et al., 2007; Liang et al., 2007; Maretschek et al., 2008; Quaglia, 2008; Porter et al., 2009). Nanotechnology is a promising field, but requires much more research to keep production costs to the minimum. Although the polymers used may be classified as safe, all encapsulated material would have to pass stringiest safety tests, which includes degradation and toxicological studies.

Conventional electrospinning gave rise to the development of co-axial electrospinning, emulsion electrospinning, and electrospinning with dual spinnerets. In co-axial electrospinning dedicated needles are used to produce the core and outer shell of the nanofibers (Sun et al., 2003). Bioactive materials (e.g., antibiotics or growth factors) are usually trapped in the core. Core-shell structured nanofibers containing bacteriocins are produced using emulsion electrospinning (Xu et al., 2006). In this case, an aqueous solution of the bacteriocin is emulsified in an organic polymer solution and then spun to encapsulate molecules in the aqueous phase (Maretschek et al., 2008).

Bi et al. (2011) extended the antimicrobial activity of nisin by at least 40 days by stabilizing the peptide in a combination of phytoglycogen octenyl succinate and oil. Similar results were reported when bacteriocin-like inhibitory substances (BLIS) and nisin were encapsulated into liposomes (Messi et al., 2003; da Silva Malheiros et al., 2012). In another study, bacteriocins of *L. salivarius*, *Streptococcus cricetus*, and *E. faecalis* were more effective than rifampicin against *M. tuberculosis* when encapsulated in liposomes (Sosunov et al., 2007). There is also a lozenge, Bactoblis^®^ (PharmExtracta^®^, Pontenure, Italy) in which bacteriocins produced by *S. salivarius* have been encapsulated.

## Animals and the Origins of Coronaviruses

The origin of SARS-CoV-2 is highly debated (Guo et al., 2020; Paraskevis et al., 2020; Wan et al., 2020). The genome of SARS-CoV-2 has a 96.3% similarity to that of BatCoV RaTG13, isolated from a bat (*Rhinolophus affinis*) and 91.02% identity with the genome of Pangolin-CoV, isolated from Malayan pangolins (Liu et al., 2019; Meo et al., 2020; Yang et al., 2020). It has been concluded from these findings that SARS-CoV-2 is genetically not closely similar to the viruses isolated from the two animals. However, this does not rule out the possibility that genetic changes could have occurred if these viruses had been transferred to humans via an intermediate host. It is thus possible that the original virus (progenitor of SARS-CoV-2) could have mutated during circulation amongst animals and developed new genetic and phenotypic properties facilitating the infection of humans (Andersen et al., 2020). This hypothesis is supported by two separate sequential outbreaks; SARS-CoV in 2002 and MERS-CoV in 2015 (Bradley and Bryan, 2019; Choudhry et al., 2019; Cui et al., 2019). If this is indeed the case, should we now anticipate that a mutant of SARS-CoV-2 may before too long fuel a sequel to the COVID-19 pandemic (COVID-20, perhaps?).

To the best of our knowledge, no systematic attempts have been made to prevent the spread of epizootic viral infections amongst bats, despite the significant number of reports describing these animals as perfect “generators” of new species/varieties of highly pathogenic viruses (Ge et al., 2013; Ithete et al., 2013; Menachery et al., 2015). In the majority of infected mammals, viruses counteract innate antiviral responses and, at the same time, activate pro-inflammatory cytokines that could lead to immunopathological disorders with lethal consequences (Tseng et al., 2012; Collins and Mossman, 2014). This is, however, not the case in bats, as they have limited ability to generate inflammatory responses to microbial infections and are pathologically asymptomatic. Bats are thus ideal “incubators” for the genetic selection of viruses and their rapid transmission to other animals, including humans (Banerjee et al., 2020).

Farm-raised and wild animals, being susceptible to viral infections, could serve as intermediary hosts, transferring viruses to other animals including humans (for review see: Malik, 2020). Viverrids could also act as hosts to SARS-CoV-2, as they have been shown to transfer other viruses between animals (Mendenhall et al., 2016; Ng et al., 2016). Three of the 10 or more types of viruses isolated from viverrids have been classified as zoonotic pathogens (including SARS-CoV) that are transmissible amongst dogs and cats (Wicker et al., 2016). Viverrids are often kept as pets, or used for the production of civet coffee, known as kopi luwak (D’Cruze et al., 2014). In some parts of the world, camels are used instead of common farm animals for transport, meat, and milk production (Widagdo et al., 2019). Zhu et al. (2019) have identified 37 different diseases in humans that originated from camels. Thirteen of these diseases had a viral etiology, with the most commonly occurring being MERS-CoV. Other viral zoonoses, such as Rift Valley fever, camelpox, hepatitis E, novel coronaviruses (HCoV-229E and UAE-HKU23), Alkhurma hemorrhagic fever, and Crimean-Congo hemorrhagic fever are becoming more prevalent in Europe and Asia. Apart from the viruses causing these symptoms, little is known about their transmission routes and molecular mechanisms (Zhu et al., 2019). Some of these viruses may very well be strong potential candidates for future pandemics.

Domestic animals may also be carriers of SARS-CoV-2. Zhang et al. (2020) have shown that cats in Wuhan (China) may have been infected with SARS-CoV-2. Fifteen of 102 cat serum samples taken during the COVID-19 outbreak were positive for the receptor-binding domain of SARS-CoV-2. Serum samples of 39 cats taken before the COVID-19 outbreak (from March to May 2019) were negative (Zhang et al., 2020). On the other hand, according to Temmam et al. (2020), domestic cats and dogs living in close contact with infected owners tested negative for SARS-CoV-2, and no antibodies against the virus were detected. Clearly, more research needs to be conducted, especially on cats. Shi et al. (2020) have shown that, from many different species of domestic animals, only cats were susceptible to SARS-CoV-2. It should, however, be pointed out that these experiments were conducted under laboratory conditions, and there is still no unequivocal proof that domestic animals are natural carriers of SARS-CoV-2.

Considering recent USDA reports^2^ ^,3^, there is an urgent need for full-scale case-control research on the possibility of cats being hosts for SARS-CoV-2 and the probability of cat-to-cat, human-to-cat, and cat-to-human transmission routes. Right now, we speculate that felines are most likely “dead-end hosts” for SARS-CoV-2 and do not pose any threat to humans. In addition, recently, minks were reported as carrying SARS-CoV-2 and may possibly be spreading the virus^4^ ^,5^.

Xia (2020) has suggested that dogs may be progenitor hosts, and possibly natural carriers of SARS-CoV-2. The authors speculated that the selection of naturally occurring mutations could have led to a reduction of CpG in SARS-CoV-2 RNA in response to the higher levels of zinc finger antiviral protein present in the gastrointestinal tracts of canines (Xu et al., 2020). However, when discussing their data, the authors did not take into consideration a previously published report by Shi et al. (2020), showing the absence of SARS-CoV-2 in dogs, even after intranasal inoculation with an extremely high titer preparation of the virus.

## Probiotics and Prophylaxis of Viral Diseases

It is becoming increasingly evident that the variety, extent, and significance of viral zoonotic infections is far greater than has been previously recognized. The scientific community, healthcare, and animal welfare organizations must now prioritize the development of acceptable and effective new strategies to reduce the genesis and transmission of these infections. This is where the use of probiotics could provide an effective prophylactic measure. Some bacteriocin-producing probiotics, including strains known to exhibit antiviral activity, have been used to affect modulation of the microbiota in livestock animals (Mingmongkolchai and Panbangred, 2018). The results of a pilot study by Meazzi et al. (2019) provide new insights into the feline gut microbiota. It was discovered that the lactobacilli present in healthy cats, were not present in animals having infectious peritonitis, caused by feline coronavirus (Meazzi et al., 2019). This points to a possible antagonistic relationship between lactobacilli and these viruses. Aboubakr et al. (2014) have also reported upon the antiviral activity of probiotic LAB against feline calicivirus (Aboubakr et al., 2014) and Stoeker et al. (2013) observed improved intestinal homeostasis in cats infected with feline immunodeficiency virus following oral administration of *L. acidophilus* (Stoeker et al., 2013). These findings all support the antiviral activity of probiotics in cats.

In other studies, *L. casei* was used as an antigen carrier to prevent diarrhea in bovines (Wang Y. et al., 2019), and a combination of probiotics was used to study the humoral response in cattle vaccinated against rabies (Vizzotto-Martino et al., 2016). Other studies have reported beneficial outcomes from the prophylactic dosing of probiotics in pigs infected with vesicular stomatitis virus, influenza A virus, transmissible gastroenteritis virus, epidemic diarrhea virus, and rotaviruses (Wang et al., 2013, 2017; Sirichokchatchawan et al., 2018; Peng et al., 2019). Clearly, the close similarities in the anatomy and physiology of humans and pigs suggest an increased potential threat of the development of suid zoonoses (Wang et al., 2013).

Additionally, there are still no effective prophylactic and treatment schedules in place for a number of viral diseases of farm animals that currently appear unlikely to become a source of human infections. Rabbit hemorrhagic disease, along with myxomatosis, is amongst the most contagious and lethal of viral infections. The gold standard for prevention of the above-mentioned diseases is vaccination; however, there are potential problems associated with its implementation, especially in relation to biosafety. In order to develop new protocols for the control of the rabbit hemorrhagic disease, Wang L. et al. (2019) suggested the use of an oral *L. casei* probiotic vaccine as an antigen delivery system to stimulate humoral immune responses against caliciviruses (Wang L. et al., 2019). However, there are currently no published studies on the use of probiotics to afford protection against myxomatosis. This indicates an opportunity for future research on this topic.

Probiotics are widely used in the poultry industry and have become a routine infection preventative in some large commercial populations. Details of the antiviral mechanisms of probiotics have, in many cases, yet to be elucidated (Dong et al., 2020). Recently, Shojadoost et al. (2019) discovered that probiotic lactobacilli stimulate cell-mediated immunity in chickens by augmenting antiviral macrophage responses. On the other hand, there also appears to be some probiotic-mediated antiviral humoral immune responses in chickens (Gonmei et al., 2019).

In aquaculture, probiotics are now also more frequently serving as preventive and management measures against a variety of microbial infections, including those that are viral. White spot syndrome virus and infectious hypodermal and hematopoietic necrosis virus have been responsible for extensive economic losses (Kuebutornye et al., 2020). A number of *Bacillus* species isolates have been used to enhance the antiviral immunity of crustaceans (Sánchez-Ortiz et al., 2016; Sekar et al., 2016; Pham et al., 2017; Kuebutornye et al., 2020).

**FIGURE 1:**
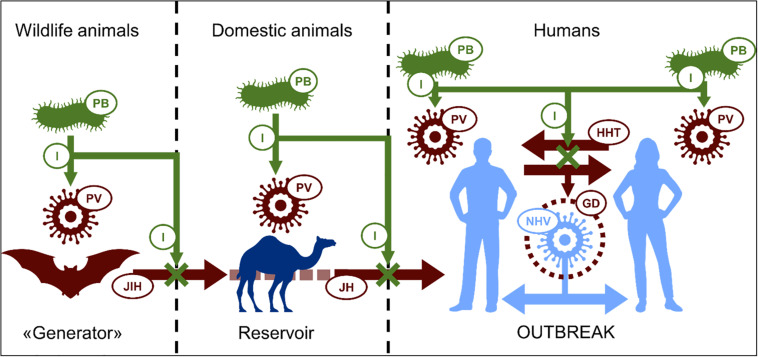
Scheme of possible mechanism of prevention of emerging viral outbreaks by probiotics: Progenitor virus (PV) developed in primary host is able to “jump” to human (JH) directly or through intermediate host (JIH – “jump” to intermediate host). Intensive human-to-human transmission (HHT) is favorable condition for PV genetic developing (GD) making it more relative to human. This in the end contributes to appearance of highly pathogenic novel human virus (NHV), causing epidemic or pandemic outbreaks. Because of antagonistic relationship, probiotic bacteria (PB) can possibly inhibit (I) virus replication in all hosts with reducing its development and transmissions, in perspective, preventing outbreaks. PV, progenitor virus; JDA, jumping to domestic animals; JH, jumping to humans; HHT, human-to-human transmission; GD, genetic development; NHV, novel human virus; P, probiotic; I, inhibition.

In some cases, it has been shown that probiotic extracellular products can help protect animals against viral infections. The antiviral activities of probiotics include proteinaceous products such as bacteriocins as well as non-proteinaceous metabolites such as lactic acid and hydrogen peroxide (Abdelhamid et al., 2019). The principal antiviral mechanisms of probiotics described to date appear to include the inhibition of virus replication (Kanmani et al., 2018). Action of this nature occurring in the initial stages of infection when virus numbers are relatively small is clearly likely to be more effective than in inflammatory stages of the infection. These observations support the contention that probiotics are, in general, more likely to be efficacious as a prophylactic measure rather than as a virus treatment. Figure 1 outlines basic strategies for the probiotic control of zoonotic viruses. Examples of viruses potentially transmissible to humans and their animal reservoirs are listed in Table 1.

**TABLE 1:** Examples of emerging zoonotic viruses.

**Animal reservoirs of virus progenitor**	**Viruses**	**Transmission route**	**References**
Bats	SARS-CoV-2	Via respiratory droplets;	Liu et al., 2019; Guo et al., 2020; Meo et al., 2020; Wan et al., 2020
Pangolins		Via contaminated surfaces	
Bats	MERS-CoV	Via consumption of products from infected camel;	Fehr et al., 2017; Bradley and Bryan, 2019; Widagdo et al., 2019; Meo et al., 2020; Yang et al., 2020
Dromedary Camels		Via contact with infected camel	
Bats	SARS-CoV	Via consumption of products from infected civet;	Wicker et al., 2017; Meo et al., 2020; Yang et al., 2020
Civets		Via contact with infected camel	
Pigs	A(H1N1)pdm09	Via respiratory droplets;	Garten et al., 2009; Watson et al., 2015; Bradley and Bryan, 2019
Birds		Via contaminated surfaces	
Bats	Ebola	Via body fluids;	Caron et al., 2018; Banerjee et al., 2020; Markotter et al., 2020
		Via respiratory droplets	
Birds	West Nile fever	Via mosquito vector	Johnson et al., 2018; Napp et al., 2018
Primates	HIV	Via sexual contacts;	Pinto-Santini et al., 2017
		Via contact with or transfer of blood, vaginal fluid, pre-ejaculate and semen	

## Can Health-Promoting Commensal and Probiotic Bacteria Defend Against Pathogenic Viruses?

The human body is extensively colonized by archaea, bacteria, fungi, and viruses, and indeed, each of us is comprised of more microbial than human cells (Sender et al., 2016a, b). Interestingly, the total number of viral particles actually exceeds the total number of microbes by around one hundred-fold. It would be naïve to think that because we can not directly visualize our microbial inhabitants, they are unlikely to be interacting with one other. Viral-bacterial interactions have long been considered exclusively as an interaction between bacteria as prey and phage as the ultimate predator. However, even phage-bacterial relationships no longer appear to be so simplistic. More likely, phages and bacteria are involved in a complex and mutually beneficial relationship allowing them both to proliferate at the site of bacterial colonization. It is also rather simplistic to view bacteria merely as objects of prey, hunted by the virus without any tools to retaliate. Among these tools are the bacteriocins, small proteinaceous anti-competitor molecules deployed by bacteria to combat excessive proliferation by their neighbors. Knowing that evolutional developments are generally economical, it is logical to suspect that bacteriocins are also antiviral weapons of bacteria. In recent decades, the use of bacteriocins as alternatives or supplements to traditional antimicrobial, antifungal, and antiviral drugs has been increasingly investigated. Many positively charged cationic bacteriocins kill susceptible bacteria by membrane permeabilization of the target cells. Antimicrobial peptides of bacterial origin play a significant role in the maintenance of stable bacterial consortia by decreasing the total number of bacterial cells in the population and preventing incursions by heterogenous microorganisms into their pre-occupied ecological niche. On the other hand, why shouldn’t virions also be a bacteriocin target? Indeed, evidence has been found for nisin activity against lactobacillus bacteriophage c2 via disruption of the viral capsid (Ly-Chatain et al., 2013). Also, coliphage can be inhibited by staphylococcin18, enterocins AAR-71, AAR-74, and erwiniocin NA4 (Qureshi T. A. et al., 2006). Not surprisingly, numerous examples of bacteriocin activity against eukaryotic viruses have also been reported (Table 2).

**TABLE 2:** Anti-viral activity of bacteriocins.

**Bacteriocin name**	**Producing strain**	**Antiviral activity tested**	**Mechanisms of action**	**References**
Enterocin CRL35	*Enterococcus mundtii* CRL35	HSV-1 and HSV-2	A late step of virus multiplication is hindered by the prevention of mainly late glycoprotein D (gamma protein) synthesis. Virus adsorption and penetration are not affected.	Wachsman et al., 1999, 2003
Enterocin ST4V	*E. mundtii* ST4V	HSV-1 and HSV-2, Poliovirus PV-3, Measles virus (strain MV/BRAZIL/001/91, an attenuated strain)	The HSV-1 and HSV-2 replication is inhibited.	Todorov et al., 2005
			Mechanism also might involve aggregation of the virus particles or blocking of their receptor sites.	
Staphylococcin 188	*Staphylococcus aureus* AB188	Newcastle disease virus	Unknown	Saeed et al., 2004
Enterocin B	*E. faecium* L3	HSV-1	Unknown	Ermolenko et al., 2010
Enterocin ST5H	*E. faecium* ST5Ha	HSV-1	Unknown	Todorov et al., 2010
Labyrinthopeptin A1	*Actinomadura namibiensis* DSM 6313	HIV-1 and HSV-1	LabyA1 interacted with envelope proteins, but not with the cellular receptors and acts as an entry inhibitor.	Férir et al., 2013
Subtilosin A	*Bacillus subtilis* KATMIRA 1933	HSV-1 and HSV-2	Acts on enveloped viruses, no activity on non-enveloped viruses. Most likely, inhibits late stages of protein synthesis. Also active against drug-resistant HSV-1.	Torres et al., 2013; Quintana et al., 2014
Enterocin B	*E. faecium* L3	À/Perth/16/2009(H3N2) and A/South Africa/3626/2013(H1N1) pdm influenza viruses	Unknown	Ermolenko et al., 2018
Bacteriocin-containing cell free supernatant	*Lactobacillus delbrueckii*	Influenza virus A/chicken/Germany, strain Weybridge (H7N7) and strain Rostock (H7N1) in cell cultures of chicken embryo fibroblasts (CEF)	Reduces expression of viral glycoproteins hemagglutinin, neuraminidase, and nucleoprotein on the surface of infected cells, reduces virus-induced cytopathic effect, infectious virus yield, and hemagglutinin production. Crude bacteriocin-containing preparation did not protect cells from infection, did not affect adsorption, and slightly inhibited viral penetration into infected cells.	Serkedjieva et al., 2000
Computer modeling Nisin- and subtilosin-derivatives	*In silico* design	Hepatitis E virus (HEV)	Theoretical estimation: binding with the capsid protein.	Quintero-Gil et al., 2017
Semi-purified bacteriocins	*Lactococcus lactis* GLc03 and GLc05, *E. durans* GEn09, GEn12, GEn14 and GEn17	Herpes simplex virus 1 (HVS-1) and Poliovirus (PV-1)	Antiviral activity before virus adsorption was recorded against HSV-1 35 for GEn14 (58.7%) and GEn17 (39.2%). Antiviral activity after virus 36 adsorption was identified against PV-1 for GLc05 (32.7%), GEn09 (91.0%), GEn12 (93.7%) 37 and GEn17 (57.2%), and against HSV-1 for GEn17 (71.6%).	Quintana et al., 2014
			The inactivation of HVS-1 viral particles may have occurred due to its interaction with the phospholipids on the viral envelope, avoiding its binding to cell receptors.	
			The inhibition of PV-1 did not occur before its adsorption.	
Duramycin	*Streptomyces cinnamoneus*	Zika virus	(An inhibitor of TIM1 receptor)	Tabata et al., 2016
			Duramycin, a peptide that binds phosphatidylethanolamine in enveloped virions and precludes TIM1 binding, reduced ZIKV infection in placental cells and explants.	
		West Nile, dengue and Ebola viruses	Inhibits the entry of West Nile, dengue, and Ebola viruses. The inhibitory effect of duramycin is specific manner: it inhibits TIM1-mediated, but not L-SIGN-mediated, virus infection, and it does so by blocking virus attachment to TIM1.	Richard et al., 2015
Micrococcin P1	*Staphylococcus equorum* WS2733	Hepatitis C	Inhibited HCV entry in a pan-genotypic manner, and prevented cell-to-cell spread without affecting the secretion of infectious HCV particles. In addition, micrococcin P1 acted synergistically with selected HCV inhibitors, and could potentially be used as a cost-effective component in HCV combination therapies.	Lee et al., 2016
Nisin	*Lactococcus lactis* subsp. *lactis*	Bovine viral diarrhea virus (BVDV)	Nisin decreased both the extracellular virus titre and theamount of intracellular viral RNA. The best effect was	Małaczewska et al., 2019
			observed when nisin was present throughout the entire	
			duration of viral infection (adsorption + post-adsorption).	
		Cytomegalovirus	Unknown	Beljaars et al., 2001
		Bacteriophage c2 (DNA head and non-contractile tail) infecting *Lactococcus* strains	The positively charged compounds can adsorb on viral capsid by also electrostatic interaction which inhibit viral adsorption on host cells.	Ly-Chatain et al., 2013
Staphylococcin 18 enterocins AAR-71, AAR-74, and erwiniocin NA4	*Staphylococcus aureus* AB188 *E. faecalis/*BLIS *Erwinia carotovora* NA4/BLIS	Coliphage HSA	Unknown	Qureshi H. et al., 2006

Although there is growing evidence documenting the action of bacteriocins against viruses, the details of the mechanism(s) of action remain largely ill-defined. Included amongst the possible suggested mechanisms are: (a) direct aggregation with viral particles thereby preventing viral entry in host cells; (b) inhibition of synthesis of viral structural proteins; (c) neutralization of virus entry due to the blocking of receptor sites on the host cell (Wachsman et al., 2003; Todorov et al., 2005; Torres et al., 2013), and (d), disruption of the capsid or supercapsid structure by the bacteriocins. Direct virucidal activity was shown for subtilosin, which inactivated HSV-1 viral particles at the non-cytotoxic concentration of 200 lg/mL. Interestingly, at lower non-virucidal levels, subtilosin inhibited the HSV-1 multiplication cycle in a dose-dependent manner (Torres et al., 2013). The peptide duramycin has been shown to have a rather specific antiviral effect: it binds to phosphatidylethanolamine in enveloped virions, precluding virus attachment to TIM1 receptors on the host cells and reducing TIM1-mediated, but not L-SIGN-mediated, virus infection (Richard et al., 2015; Tabata et al., 2016). Labyrinthopeptin A1 (LabyA1) also acts as an entry inhibitor against HIV and HSV. It was revealed that LabyA1 interacted with the HIV envelope protein gp120, but not with HIV cellular receptors (Férir et al., 2013).

The hypothesis that bacteriocins as positively charged compounds can adsorb to viral capsids by electrostatic interaction, thereby inhibiting viral adsorption to host cells was tested in a bacteriophage-based test system (Ly-Chatain et al., 2013). Wachsman et al. (2003) showed that the inhibition of HSV replication by enterocin CRL35 was due to interference with intracellular viral multiplication via the prevention of glycoprotein D (gamma protein) synthesis. HSV adsorption and penetration were not affected. Bacteriocin ST4V, from *E. mundtii*, inhibited both HSV-1 and HSV-2 replication in a dose-dependent manner (Todorov et al., 2005).

This growing evidence of antiviral activity associated with some bacteriocins supports speculation that the beneficial biological attributes of probiotics are not limited to the previously well-documented evidence of their immune modulation and anti-bacterial activities. We venture to speculate that at least in part, the anti-infection activities of some probiotic bacteria might include the capability of limiting or even eliminating the replication of pathogenic viruses. The evolutionary genesis of microbes capable of carrying out these actions appears quite logical as a protective response of a stabilized indigenous ecosystem to potential territorial invasion regardless of whether it is bacterial, fungal, or viral in origin.

As was rightfully pointed out in a recent review by Akour (2020), presently, there are no published reports on the implementation of probiotics for prophylaxis or combating COVID-19. However, there is a Phase II clinical trial to evaluate the previously explored anti-asthma “live cells” formulation MRx4DP0004 as an immunomodulatory drug for hospitalized COVID-19 patients^6^. While the actual formulation is not reported, the patients received a daily dose of 4 × 10^9^ to 4 × 10^10^ live cells in two doses, twice daily for 2 weeks. It is only logical to speculate that properly selected probiotics can serve as effective adjuvants for the prophylaxis and even treatments of COVID-19. Until the pathogenesis of novel coronavirus and its effect on gut microbiota is established, the use of probiotics may not be appropriate. In addition, while foodborne transmission of COVID-19 is rather unlikely (Li et al., 2021), appropriate model studies should be conducted to exclude food as a possible vehicle for the virus transmission. Since immune-compromised humans are likely to be most vulnerable to COVIV-19, the use of probiotics as prophylactic agents or adjuvants in the treatment regiment should be carefully considered, bearing in mind that even the friendliest, most studied probiotics may cause septicemia in immune-compromised and otherwise challenged health status individuals (Kochan et al., 2011; Kulkarni and Khoury, 2014; Doron and Snydman, 2015; Koyama et al., 2019).

## Conclusion

Probiotics have been defined and are now ubiquitously referred to as “live microorganisms that, when administered in adequate amounts, confer a health benefit on the host” (Hill et al., 2014). More recently, however, attention has been drawn to the frequent misapplication of the term “probiotic” to describe any microbe having plausible therapeutic utility in the human host (Reid et al., 2019). Indeed, although the notion that ingesting or administering live microbes can achieve beneficial therapeutic outcomes is gaining widespread public acceptance, many studies still “overstate” the importance of their scientifically tenuous findings (Hooks et al., 2018). From a historical perspective, various strains of lactobacilli and bifidobacteria have had long-standing *ad hoc* traditional therapeutic applications for the treatment of infections of the gastrointestinal tract and vagina. For some time following the advent of the antibiotic era, the probiotic usage of these bacteria decreased. However, now in the face of escalating antibiotic resistance and a resurgence of infectious diseases in an aging and more compromised human population, ever-increasing attention is being given to the plausible use of probiotics to supplement or even to supplant existing therapies. Key players in the contemporary probiotic team include the LAB, the bifidobacteria, enterics, and commensal streptococci. Amongst the more commonly touted attributes of successful probiotic microbes are:

(1) Host-beneficial processing of cellular products or dietary constituents;
(2) Appropriately targeted and suitably controlled production of antimicrobial activity that is capable of reequilibrating dysbiotic populations of indigenous and immigrant prokaryotes, archaea, and viruses associated with human or other animal hosts;
(3) Activities directed against eukaryotic cells ranging from limiting the growth of parasitic fungi and protozoa to the subtle modulation of host cell functions, especially within the immune system, but also including other cell signaling functions.

Some probiotics stimulate genes involved in the attenuation of the pro-inflammatory response or in the promotion of homeostasis (Cosseau et al., 2008). The efficacy of a probiotic depends on its metabolic characteristics, the molecules presented on its surface, and on its secreted products. Cellular components such as its DNA and peptidoglycan may also be influential.

The contemporary availability and application of ever more sophisticated technologies is now revealing that all bacteria (including probiotics) likely contain multiple genetic loci encoding for the production of (and self-protection against) antimicrobial substances. Indeed, the production of these molecules is an essential survival characteristic for any microbe contemplating sustainable existence within a heterogeneous microbiota. They can function as: (a) colonization aids – facilitating the entry of the producer strain into an existing niche already occupied by other microbes; (b) killing peptides – inhibitory to the proliferation of competitors; (c) signaling peptides – influencing other bacteria via quorum sensing and bacterial cross-talk in heterogeneous communities or by signaling to cells of the host’s immune system. Bacteriocins can function as sensors for the immune system, indicating new bacterial challenges are occurring (Dobson et al., 2011b). Some bacteriocins function as inhibitory molecules at high concentrations and as signaling molecules at low levels (Fajardo and Martínez, 2008). Another function is that of auto-inducing peptides to boost the competitive efficacy of the producer bacterium. Included amongst these anti-competitor molecules are the following:

(a) Relatively non-specifically toxic low molecular weight metabolites.
(b) Proteinaceous molecules, some of which are initially referred to as bacteriocin-like inhibitory substances (or BLIS), but when fully characterized have been found to display a remarkable diversity of molecular identities. Their prolific existence is a reflection of their functional importance to the bacterial inhabitants of complex natural ecosystems. The effector molecules have a variety of designations ranging from the directly ribosomally synthesized bacteriocins to ribosomally synthesized and post-translationally modified peptides (RiPPs), many of which are now being initially discovered using *in silico* methods.

The contemporary anti-infection or microbiota-modulation applications of probiotics fall loosely into two broad categories – (a) prophylaxis: regular low-level dosing is used with the intention of maintaining an ongoing presence (and efficacy) of the probiotic within the host’s microbiota; and (b) therapy: relatively high-level dosing is directed either to the site of infection, against dysbiosis of the microbiota as a whole, or to favor interaction with immunologically responsive host tissues. The idea of using probiotics or their extracellular products to control viral infections is a recent concept. Further research is required to understand possible chemical or physical interactions between probiotic cells (or their metabolites) and virions, possible interference with virus attachment sites, replication, and the role probiotics have on our immune system when fighting viral infections. Will these strategies find application as stand-alone antiviral prophylactic or therapeutic interventions, or will some be developed for use in synergistic combinations with conventional antiviral agents? These questions will help researchers find solutions for existing viral infections causing lethal diseases, including COVID-19. The recent findings recommend the use of probiotics for modulation of gut microbiota in respiratory infections suggesting their possible applications in the management or treatment of COVID-19.

**FIGURE 2:**
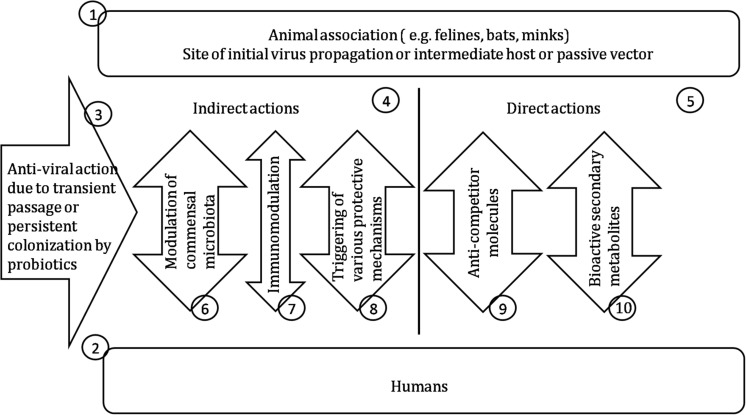
Probiotics for control of viral infections. (1) Either the original virus source or a vector functioning as alternative replication host or for mechanical transfer of the virus. (2) The newly acquired host for virus proliferation. (3) Probiotic intervention may be either short –term or enduring depending on the colonization efficacy of the probiotic. (4) Boosting of the host’s own tissue, immunological or microbiota defenses following interaction with the probiotic. (5) Direct antiviral activity of probiotic cellular components or extracellular products. (6) Temporary or persisting microbiota changes following probiotic exposure associated with enhanced antiviral activity. (7) Boosting the innate antiviral immune defenses. (8) Up-regulating antiviral defenses of non-immunological body components. (9) Probiotic products having anti-competitor roles such as bacteriocins and ribosomally synthesized, posttranslationally modified peptides (RiPPs). (10) Probiotic metabolites detrimental to virus replication.

Thus, probiotics and their metabolites clearly have considerable potential to effect “germ warfare” against disease-causing viruses (Figure 2) and also to be key allies in our efforts to reduce the usage of toxic virucidal chemical agents. Although the detailed mechanism(s) by which probiotics can restrict viral multiplication are not yet fully elucidated, the attention of the scientific and medical communities is now becoming increasingly focused upon the benefits to be had from backing the probiotics in the war against viruses. We hope that this review may direct attention toward the unknown aspects of antiviral probiotics, highlight important unanswered questions, and serve as a springboard for the further development of novel probiotic based therapies to effectively combat emerging zoonotic viral threats.

## Author Contributions

All authors listed have made a substantial, direct and intellectual contribution to the work, and approved it for publication.

## Conflict of Interest

JT was employed by BLIS Technologies Ltd. The remaining authors declare that the research was conducted in the absence of any commercial or financial relationships that could be construed as a potential conflict of interest.

## Footnotes

1 https://www.worldometers.info/coronavirus/?utm_campaign=homeADemocracynow(2020)%20dvegas1? (accessed 20 April 2020).

2 https://www.aphis.usda.gov/aphis/newsroom/news/sa_by_date/sa-2020/sars-cov-2-animals

3 https://www.aphis.usda.gov/aphis/newsroom/news/sa_by_date/sa-2020/ny-zoo-covid-19

4 https://www.rijksoverheid.nl/actueel/nieuws/2020/04/26/covid-19-geconstateerd-op-twee-nertsenbedrijven

5 https://www.rijksoverheid.nl/documenten/kamerstukken/2020/04/26/kamerbrief-infectie-van-nertsen-met-sars-cov-2

6 https://clinicaltrials.gov/ct2/show/NCT04363372
